# Augmented and Virtual Reality in Cosmetic Dermatology

**DOI:** 10.1111/jocd.16698

**Published:** 2024-11-21

**Authors:** Marina Landau, Maria Tsoukas, Mohamad Goldust

**Affiliations:** ^1^ Arena Dermatology and Department of Plastic Surgery Shamir Medical Center Be'er Ya'akov Israel; ^2^ Department of Dermatology University of Illinois College of Medicine at Chicago Chicago Illinois USA; ^3^ Department of Dermatology Yale University School of Medicine New Haven Connecticut USA

**Keywords:** augmented reality, cosmetic dermatology artificial intelligence, metaverse, virtual reality

Dear Editor,

Augmented reality (AR) and virtual reality (VR) have the potential to transform cosmetic dermatology, evolving from theoretical concepts to essential tools in patient care [1, 2]. AR incorporates digital elements into the real world, while VR engages users in simulated environments, enabling new ways of visualizing treatments and refining both patient and practitioner experiences. These technologies are used to improve procedural accuracy, enhance training, and optimize patient outcomes [3].

AR can be a key resource in treatment planning and application, particularly for minimally invasive procedures such as dermal fillers. By overlaying digital images onto a patient's skin in real time, AR allows dermatologists to visualize projected outcomes before any intervention is performed. This ensures that treatment plans are not only precise but also personalized to each patient. The integration of artificial intelligence (AI) into AR enhances this capability by providing accurate predictions of results. AI‐based systems will guide practitioners in selecting the appropriate number of syringes, types of fillers, injection techniques, and toxin units, while also predicting outcomes for energy‐based devices like lasers. This allows for customized treatment plans, reducing the likelihood of over‐ or under‐treatment and enhancing patient satisfaction [4].

Moreover, AR's role during procedures ensures greater accuracy by assisting dermatologists with real‐time visual cues for injection sites, toxin placement, and filler distribution. Such precision lowers the risk of complications and ensures that treatments meet patient expectations.

On the other hand, VR plays a crucial role in patient education and experience. Patients who may feel anxious about undergoing cosmetic treatments can benefit from VR simulations that walk them through the procedural steps, helping them set realistic expectations for recovery and results. For example, a patient considering laser resurfacing or chemical peels can experience a virtual procedure, gaining clarity on pre‐treatment preparation, procedure steps, and post‐operative care. This huge experience provides trust and reduces anxiety, ensuring patients make informed decisions [5].

VR also offers a risk‐free, tactile training environment for practitioners. Dermatologists can practice complex procedures, such as laser treatments or microneedling, receiving real‐time feedback on their actions. This enhances skills and confidence, ensuring that trainees are well‐prepared before treating real patients. In addition, VR can aid in consultations, enabling patients to visualize various treatment outcomes. This personalized approach ensures that the selected treatments match with the patient's facial structure and aesthetic goals.

However, challenges remain. The high cost of implementing AR and VR technologies and the need for specialized training could block widespread adoption. These technologies also require multidisciplinary collaboration, particularly in fields like transgender medicine, where precision and personalization are important. Moreover, issues of data privacy, ethics, and security must be addressed to protact patient information and build trust in these tools.

Relying on AI‐based predictions can lead to incorrect assessments and treatment outcomes if the algorithms do not accurately consider individual patient variables. Such errors may stem from biases in training data, over‐reliance on generalized models, or technological malfunctions. In some cases, this can result in over‐treatment, under‐treatment, misapplication of fillers or toxins, and unexpected adverse effects that reduce patient satisfaction. As these technologies become more integrated into healthcare, it is essential to establish protocols for reviewing and verifying AI‐generated recommendations. This will help prevent harm and ensure that AI assistance aligns closely with each patient's unique needs.

In conclusion, AR and VR have the potential to expand cosmetic dermatology by enhancing patient education, improving procedural accuracy, and optimizing practitioner training. Despite the challenges of cost, access, and data privacy, these technologies are set to become integral in modern dermatologic practice as they continue to evolve.

## Consent

Informed consent is unnecessary for this review.

## Conflicts of Interest

The authors declare no conflicts of interest.

## Data Availability

Data sharing not applicable to this article as no datasets were generated or analysed during the current study.
